# Stromal-Cell and Cytokine-Dependent Lymphocyte Clones Which Span the Pre-B- to B-Cell Transition

**DOI:** 10.1155/1991/79721

**Published:** 1991

**Authors:** Katsuhiko Ishihara, Kay Medina, Shin-Ichi Hayashi, Carolynn Pietrangeli, Anthony E. Namen, Kensuke Miyake, Paul W. Kincade

**Affiliations:** Oklahoma Medical Research Foundation 825 N.E. 13th Street Oklahoma City Oklahoma 73104 USA

## Abstract

Five stromal-cell-dependent lymphocyte clones are described that correspond to late pre-B or early B-cell stages of differentiation.They are useful for determining the molecular requirements for pre-B replication, for studying the stromal cells that supply those factors, and for delineating the final sequence of differentiation events as newly formed lymphocytes prepare to exit the bone marrow. The efficiency of lymphocyte growth at limiting dilution varied substantially on different stromal-cell clones and may reflect functional heterogeneity of stromal cells. Most lymphocyte clones were similar to uncloned lymphocytes from Whitlock-Witte cultures in that they responded only transiently to interleukin-7 (IL-7) and then died, unless maintained on a stromal-cell clone. One unusual lymphocyte clone (2E8) was propagated for more than 1 year in IL-7 alone and was selectively responsive to that cytokine. Most of the lymphocyte clones were not tumorigenic in immunodeficient mice. However, one pre-B clone (1A9)’grew autonomously in culture when held at high density, responded to conditioned medium from a number of cell lines, and was tumorigenic. Tumors derived from this clone were infiltrated by stromal cells and lymphocytes taken from the tumors' retained characteristics of the original clone. Ly-6 antigens were inducible on 2E8 and 1A9 cells, but the lymphocytes were otherwise arrested in differentiation. The 2E8 cells had rearranged and expressed κ light-chain genes but displayed them on the surface along with surrogate light chains and μ heavy chains. Thus, expression of authentic Tight chain need not coincide with termination of surrogate light-chain utilization in newly formed B cells. Several glycoproteins have recently been demonstrated to be associated with surface immunoglobulin (Ig) on mature B-lineage cells and plasma-cell tumors. We now show that one member of this family (approximately 33 kD) was associated with the μ+surrogate light-chain complex on the 1A9 pre-B-cell clone. When compared to mature B lymphomas, fewer bands coprecipitated with the surface-labeled Ig isolated from pre-B- and early B-cell lines, suggesting that components of the antigen receptor are sequentially acquired during development. The normal replication and differentiation of pre-B cells is probably regulated by complex interactions with multiple cytokines and matrix components of the marrow microenvironment. Cloned lymphocyte lines that are dependent on stromal cells should continue to be important tools for molecular definition of those interactions.


*Developmental Immunology*, 1991, Vol. 1, pp. 149–161
Reprints available directly from the publisher
Photocopying permitted by license only

© 1991 Harwood Academic Publishers GmbH
Printed in the United Kingdom

### Stromal-Cell and Cytokine-Dependent Lymphocyte Clones Which Span the Pre-B- to B-Cell Transition

KATSUHIKO ISHIHARA, KAY MEDINA, SHIN-ICHI HAYASHI, CAROLYNN PIETRANGELI, ANTHONY E. NAMEN, KENSUKE MIYAKE and PAUL W. KINCADE*

*Oklahoma Medical Research Foundation, 825 N.E. 13th Street, Oklahoma City, Oklahoma 73104*

Five stromal-cell-dependent lymphocyte clones are described that correspond to late pre-B or early B-cell stages of differentiation. They are useful for determining the molecular requirements for pre-B replication, for studying the stromal cells that supply those factors, and for delineating the final sequence of differentiation events as newly formed lymphocytes prepare to exit the bone marrow. The efficiency of lymphocyte growth at limiting dilution varied substantially on different stromal-cell clones and may reflect functional heterogeneity of stromal cells. Most lymphocyte clones were similar to uncloned lymphocytes from Whitlock-Witte cultures in that they responded only transiently to interleukin-7 (IL-7) and then died, unless maintained on a stromal-cell clone. One unusual lymphocyte clone (2E8) was propagated for more than 1 year in IL-7 alone and was selectively responsive to that cytokine. Most of the lymphocyte clones were not tumorigenic in immunodeficient mice. However, one pre-B clone (1A9) grew autonomously in culture when held at high density, responded to conditioned medium from a number of cell lines, and was tumorigenic. Tumors derived from this clone were infiltrated by stromal cells and lymphocytes taken from the tumors' retained characteristics of the original clone. Ly-6 antigens were inducible on 2E8 and 1A9 cells, but the lymphocytes were otherwise arrested in differentiation. The 2E8 cells had rearranged and expressed $\kappa$ light-chain genes but displayed them on the surface along with surrogate light chains and $\mu$ heavy chains. Thus, expression of authentic light chain need not coincide with termination of surrogate light-chain utilization in newly formed B cells. Several glycoproteins have recently been demonstrated to be associated with surface immunoglobulin (Ig) on mature B-lineage cells and plasma-cell tumors. We now show that one member of this family (approximately 33 kD) was associated with the $\mu$+surrogate light-chain complex on the 1A9 pre-B-cell clone. When compared to mature B lymphomas, fewer bands coprecipitated with the surface-labeled Ig isolated from pre-B- and early B-cell lines, suggesting that components of the antigen receptor are sequentially acquired during development. The normal replication and differentiation of pre-B cells is probably regulated by complex interactions with multiple cytokines and matrix components of the marrow microenvironment. Cloned lymphocyte lines that are dependent on stromal cells should continue to be important tools for molecular definition of those interactions.

KEYWORDS: pre-B cells, surrogate light chains, Ig-associated proteins, interleukin-7.

### INTRODUCTION

Cytokine-dependent cell lines have been extremely valuable as biological indicators for investigating the growth requirements of lymphoid and hemopoietic cells. One lymphocyte clone (2E8) was isolated from murine long-term bone marrow cultures and used for the initial selection of a series of stromal-cell clones (Pietrangeli et al., 1988). We now describe four additional stromal-cell-dependent lymphocyte clones that have different growth requirements and potential for tumor formation *in vivo*. We also report that surrogate light chains and associated proteins were detectable on the surface of cells that appear to represent the final stages in B-lymphocyte formation. These long-term cultured lymphocytes may or may not have exact counterparts in normal bone marrow. Hoewever, they reveal functional hetero-

*Corresponding author.

geneity in stromal-cell clones and should be useful in determining what regulatory molecules besides IL-7 participate in B lymphopoiesis.

### RESULTS

### General Characteristics and Patterns of Gene Expression

Four new lymphocyte clones were established by limiting dilution from long-term Whitlock-Witte cultures. Three of our lymphocyte clones were derived from BALB.xid strain mice. These may be classified as immature B cells, expressing both $\mu$ and $\kappa$, as well as the CD45R epitope detected by the monoclonal 14.8 antibody (Table 1). The 1A9 lymphocyte clone was isolated from cultures derived from normal BALB/c-strain mice and it is a pre-B-cell line. A detailed characterization was then performed to determine which B-lineage genes are expressed in these clones and localize them at particular developmental stages.

TABLE 1
Characteristics of Marrow Lymphocyte Clones

| | Immunofluorescence[a] | | | | | | | |
|---|---|---|---|---|---|---|---|---|
| Clone | s$\mu$ | s$\kappa$ | s$\lambda$ | 14.8 | BP-1 | Syndecan | Ly-6 | Ia[d] |
| 2G5 | + | + | n.d.[b] | + | − | n.d. | n.d. | + |
| 3A6 | + | + | n.d. | + | − | − | − | + |
| 2H6 | + | + | − | + | (+) | − | − | ± |
| 2E8 | + | + | − | + | + | − | i | + |
| 1A9 | + | − | − | (+) | − | + | i | − |

[a]Cell-surface-marker expression was evaluated on the lymphocyte clones by staining with the indicated monoclonal antibodies. The 2H6 clone lost the BP-1 antigen with extended culture and the Ly-$5_{220}$/CD45 antigen detected by monoclonal 14.8 antibody disappeared from 1A9 cells with time. Although the Ly-6A antigen detected by monoclonal SK 70.94 antibody was not initially detectable on 2E8 or 1A9 cells, it was induced (i) by incubation with recombinant $\gamma$IFN.
[b]n.d. = not examined.

Two of the clones initially bore the BP-1 antigen, which is preferentially expressed on pre-B cells and newly formed B cells in normal bone marrow (Cooper et al., 1986; Wu et al., 1990; Table 1). Some subcultures of the 2E8 clone subsequently lost this marker. Class II antigen is normally acquired at the same time, or soon after, expression of surface Ig molecules (Kincade et al., 1981) and this was just detectable on one clone (2H6) and clearly positive on three others (2E8, 3A6 and 2G5). Only the 1A9 clone expressed the integral membrane proteoglycan Syndecan, which has previously been found on pre-B cells (Sanderson et al., 1989).

The 70Z/3 pre-B lymphoma line has been extensively studied as a model of inducible $\kappa$ light-chain expression (Paige et al., 1978). We did not find evidence for a similar response in 1A9 cells, whose light-chain genes are in germ-line configuration and the characteristics of 1A9 cells recovered from *in vivo* tumors were similar to the 1A9 culture line (see what follows). Two of the lines (2E8 and 1A9) were responsive to $\gamma$IFN and this resulted in strong expression of the Ly-6A antigen (Table 1 and data not shown). This was not seen with the 2H6 and 3A6 clones and in none of the clones did the density of class II antigens change with IFN exposure. These experiments do not rule out the possibility that some or all of the cloned cells have differentiation potential. However, they do indicate that their characteristics are relatively stable with prolonged culture. In contrast to another recently described group of lymphocyte clones (Tominaga et al., 1989), none of our clones had detectable CD5 (Ly-1) antigen. In this respect, they were like the bone marrow precursors from which they derived.

Previous studies demonstrate that expression of certain genes generally corresponds to stages of B-lineage differentiation (Sakaguchi and Melchers, 1986; Zimmerman et al., 1986; Bender and Kuehl, 1987; Park and Osmond, 1987). Selected probes were used to compare transcripts from our lymphocyte clones and several transformed cell lines by Northern blot analysis (summarized in Table 2 and selected examples shown in Fig. 1). B29 is a member of the Ig superfamily that is highly specific for B-lineage cells (Hermanson et al., 1988a) and has sequence similarity to an Ig-associated protein (Reth, 1989). It was expressed at a high level in all of these cells. More variable were levels of transcripts for B37, which is thought to encode a protein with homology to nuclear oncogenes (Hermanson et al., 1988b; Wall, manuscript in preparation). The same was true for the type II membrane protein, Lyb-2 (Nakayama et al., 1989). Only one of the new clones expressed terminal deoxynucleotidyl transferase (TdT), which is thought to be responsible for insertion of nongerm-line nucleotides into rearranging Ig genes (Desiderio et al., 1984). The $\lambda_5$ gene is thought to encode the $\omega$ surrogate light chain (Sakaguichi and Melchers, 1986; Tsubata and Reth, 1990). Although a trace was detectable in the W279 B lymphoma, levels were extremely high in the pre-B

TABLE 2
Characteristics of Marrow Lymphocyte Clones

| | Northern Blot Analysis[a] | | | | | | | | | | | |
|---|---|---|---|---|---|---|---|---|---|---|---|---|
| Clone | TdT | B37 | B29 | $\lambda_5$ | Lyb-2 | Ly-5 | BP-1 | Lck | Blk | N-Myc | Myb | $\kappa$ |
| MPC11 | – | – | +++ | – | – | – | – | – | – | – | – | ++++ |
| W279 | – | +++ | +++ | + | + | ++ | – | ++ | + | – | – | ++ |
| 3A6 | – | + | +++ | ++ | + | ++ | – | ± | – | – | + | + |
| 2H6 | + | ++ | +++ | +++ | ++ | ++ | – | ++ | – | – | + | + |
| 2E8 | – | +++ | +++ | ++++ | ++ | ++ | +++ | ++ | ++ | ++ | ++ | ++ |
| 70Z/3 | + | ++ | +++ | ++++ | + | ++ | + | ++ | + | – | ++ | – |
| 1A9 | – | + | +++ | ++++ | ++ | +[b] | + | ++ | – | + | ++ | – |

[a]Northern blots were prepared with total RNA from the lymphocyte clones, as well as several reference cell lines: MPC11 (plasmacytoma); W279 (B lymphoma); and 70Z/3 (Pre-B lymphoma). Membranes were sequentially tested with the indicated cDNA probes and the relative abundance of transcripts assessed by comparison to actin mRNA. An arbitrary scale of undetectable (–) to extremely abundant (++++) was used and the results summarized in this table. See Fig. 1 for a photograph prepared from representative blots.

[b]Most of the cell lines expressed the large transcript of Ly-5/CD45 (approximately 5 kb) usually found in B-lineage cells. An exception was 1A9, which had the short mRNA species (approximately 4.7 kb) typical of thymocytes and T lymphomas.

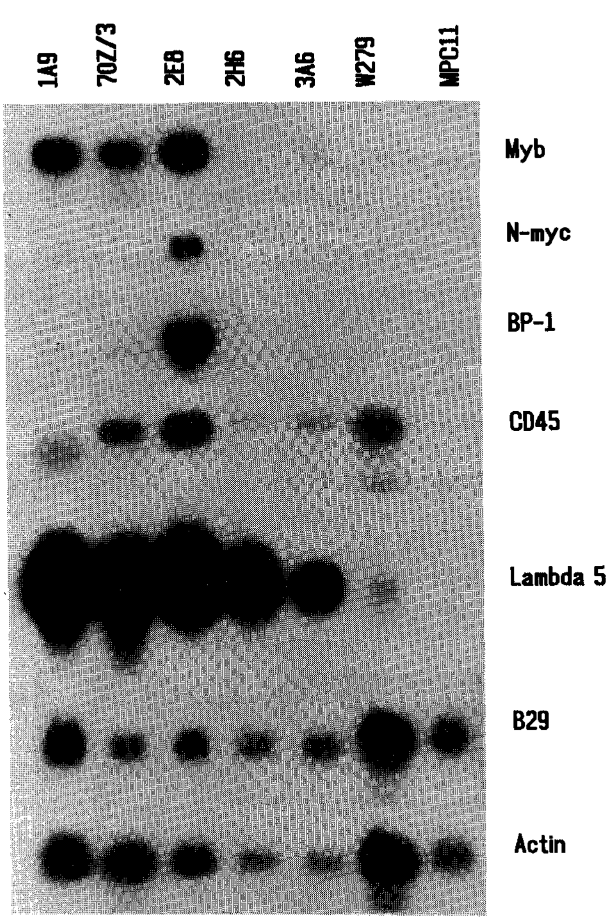

FIGURE 1. Northern blot analysis of lymphocyte clones compared to pre-B lymphoma (70Z/3), B lymphoma (W279), and myeloma (MPC11) cells. Replicate Northern blots were repeatedly hybridized, stripped, and reprobed with the indicated cDNA probes. These results are representative of a more extensive analysis, which is summarized in Table 2.

lymphoma (70Z/3) from which it was originally isolated, and our new lymphocyte clones.

The fact that many long-term cultured lymphocytes lack the CD45R epitope (Witte et al., 1986) was reflected by the loss of this marker on 1A9 lymphocytes with subculture (Table 1). It is interesting that Northern blots prepared with RNA from CD45R negative 1A9 cells had the short mRNA species typical of thymocytes, rather than the approximately 5-kb band in B cells (Fig. 1). Loss of the CD45R epitope may have resulted from a change in exon utilization (Thomas et al., 1987) with time in culture. Similarly, an endopeptidase encoded by the BP-1/6C3 gene may be lost in transformed cells that contain a shortened transcript (Wu et al., 1990). BP-1 mRNA was most abundant in 2E8 cells that were strongly positive for the BP-1 antigen (Table 2 and Fig. 1).

A lymphocyte tyrosine kinase (*lck*) was expressed by all of the lymphocyte clones, albeit in different amounts, whereas transcripts for a B-cell-specific tyrosine kinase (*blk*) were detectable only in 2E8 cells (Fig. 1). Products of these genes may be important for signal transduction in normal lymphocytes and are expressed at variable levels in established cell lines (Garvin et al., 1988; Dymecki et al., 1990).

Altered expression of two protooncogenes has been associated with the transition of pre-B cells to B cells (Zimmerman et al., 1986; Bender and Kuehl, 1987). One of our B-cell clones contained an N-*myc* transcript, consistent with its retention of most other pre-B characteristics. N-*myc* mRNA was also detectable in the 1A9 pre-B-cell clone (Fig. 1). All of our clones expressed *myb* mRNA, whose transcription level drops dramatically in newly formed B cells (Bender and Kuehl, 1987). None of the lymphocyte clones had transcripts detectable by Northern analysis for CD14, ckit, IL-6, or IL-7.

Southern blot analyses were done to determine the rearrangement status of immunoglobulin genes in several of the clones. Both alleles were rearranged with respect to heavy-chain $J_H$ segments in 1A9 and 2E8 cells. One $\kappa$ allele was rearranged in 2E8 cells, whereas neither $\kappa$ nor $\lambda$ light-chain genes were rearranged in the 1A9 pre-B-cell clone (data not shown).

On the basis of surface markers and the pattern of expression of mRNA species, we conclude that these lymphocyte clones correspond to the late pre-B- (1A9) or newly formed B-cell (2E8, 2H6, 2G5, and 3A6) stages of development. Of some 14 non-immunoglobulin genes and markers whose expression was tested, only two, Syndecan and class II antigen, consistently distinguished the pre-B- and B-cell clones.

**Light Chains and Ig-Associated Proteins**

The 1A9 lymphoycte clone had a number of interesting features. Firstly, it expressed substantial amounts of surface $\mu$ heavy chains, but was negative for conventional $\kappa$ or $\lambda$ light chains (Table 1). Rare murine and human pre-B-cell lines have been found with this characteristic and the membrane-bound $\mu$ chains were associated with one or more "surrogate" light chains (Sakaguchi and Melchers, 1986; Kudo and Melchers, 1987; Pillai and Baltimore, 1987; Kerr et al., 1989). Cell-surface glycoproteins of 1A9 cells were biotinylated, extracted, and immunoprecipitated with rabbit anti-$\mu$. In addition to the 75–85-kD $\mu$-chain band, a doublet of approximately 18 and 19 kD bands was visible when this material was subjected to polyacrylamide gel electrophoresis (Fig. 2). This was observable regardless of whether digitonin or Triton-X detergents were used (Fig. 2). Of particular interest was the finding that the more mature 2E8 cells also had these surface molecules, in

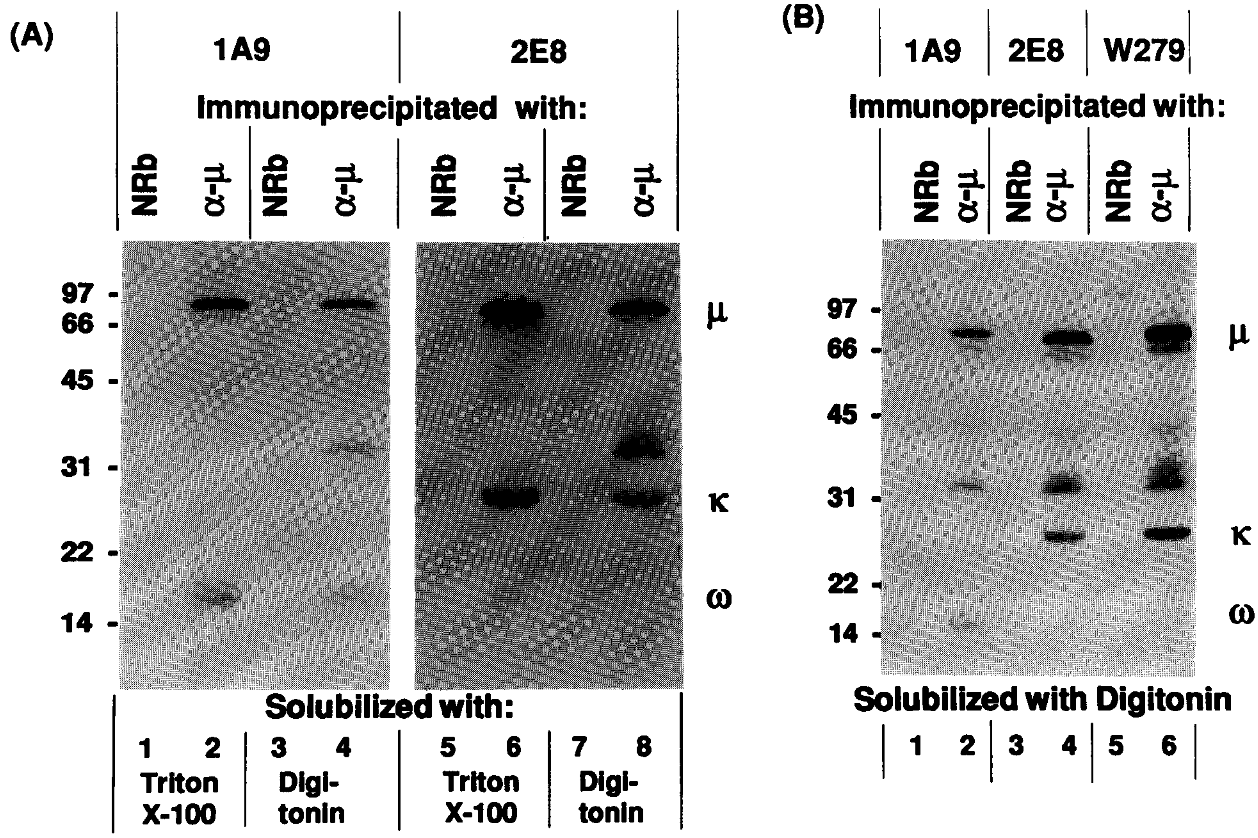

FIGURE 2. Immunoprecipitation of biotin-labeled cell-surface antigens. The indicated cell lines were surface-labeled, and solubilized with either Trion X-100 or Digitonin as indicated. Lysates were precleared with normal rabbit serum (NRb) coupled to protein G beads prior to secondary immunoprecipitation with rabbit antimouse $\mu$-chain-coupled beads. Precipitated material was analyzed by 12% (A) or 10% (B) SDS-PAGE under reducing conditions and visualized by exposure to an avidin-peroxidase conjugate.

addition to the conventional 27 kD $\kappa$ light-chain band. As noted before, the $\lambda_5$ transcript thought to encode the $\omega$ surrogate light chain was detectable in all of the lymphocyte clones.

A complex of at least three proteins has recently been discovered in association with the surface Ig of mature B cells (Haustein' and Von der Ahe, 1986; Hombach et al., 1988; Campbell and Cambier, 1990; Chen et al., 1990; Hombach et al., 1990; Wienands et al., 1990). Little is known about their utilization on pre-B cells and this question was addressed with two of our new lymphocyte clones. These proteins were detectable only in digitonin (Oettgen et al., 1986) lysates (Fig. 2A). The complex pattern of approximately 33-, 35-, and 36-kD species obtained with W279 B lymphoma cells (Fig. 1B) probably corresponds to the 32–39-kD proteins previously detected on mature IgM$^+$ B cells (Campbell and Cambier, 1990; Hombach et al., 1990). Only one clear band of approximately 35 kD was observed with extracts of 1A9 cells (Fig. 2). A slightly more complex pattern (probably reflecting the presence of 33- and 35-kD species) was consistently observed in studies of 2E8 cells (Fig. 2). Thus, Ig-associated proteins may be displayed in a sequential pattern during B-lineage differentiation.

### Growth on Cloned Stromal-Cell Lines

Cloning of lymphocytes in methyl cellulose on stromal-cell clones permits quantitative assessment of their growth requirements and provides information about the functional heterogeneity of stromal cells (Pietrangeli et al., 1988). The four new clones differed in two respects from an earlier lymphocyte clone (5A12), which was initially used for classification of our stromal-cell clones. Firstly, none proliferated on a spleen-derived stromal-cell clone (SS1) (Fig. 3). One possible explanation for this may lie in the fact that, in other studies, the spleen-derived stromal cells appeared by Northern blot analysis to elaborate little interleukin-7 (Gimble et al., 1989b). Two of the four lymphocyte clones (2G5 and 1A9) had low, but often measurable, cloning efficiencies

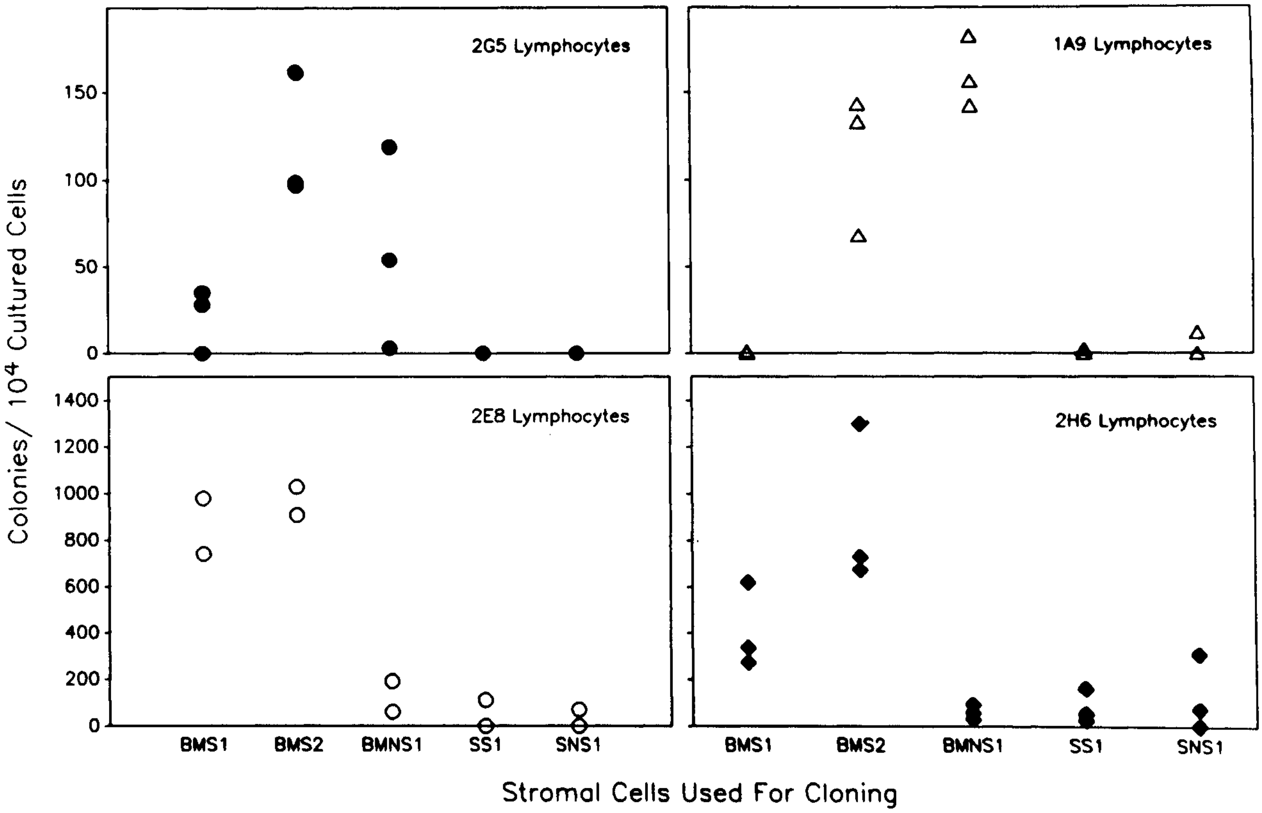

FIGURE 3. Clonal proliferation of lymphocyte clones in methyl cellulose on adherent layers prepared with different stromal cell clones. Note that different scales are used for lymphocyte clones 2G5 and 1A9, which had low cloning efficiencies relative to 2E8 and 2H6.

on the BMNS1 stromal-cell clone, but did not grow on adherent layers of BMS1. A quite different pattern was observed with the 2E8 and 2H6 lymphocyte clones. Both of them consistently cloned with high efficiency on BMS2- or BMS1-adherent cell layers. In other studies, the BMS2 stromal-cell clone has been subjected to detailed characterization because of its universal support of all lymphocyte clones (Gimble et al., 1989a; Gimble et al., 1989b; Gimble et al., 1990). The BMS1 stromal-cell clone appears to be highly selective for growth of 2E8 or 2H6, but not 1A9 lymphocytes. Correlation of the growth of these lymphocyte clones with expression of cytokine genes in the BMS1 and BMS2 cells may be informative with respect to molecular components of the bone marrow microenvironment.

### Tumorigenicity

Due to the high proliferative rate of both the lymphocyte and stromal-cell clones used in these studies, we investigated their tumorigenicity by injecting the cells subcutaneously into immunodeficient (nu/nu) or SCID mice (Table 3). None of the stromal-cell clones formed tumors under these conditions and only the 1A9 lymphocytes produced palpable nodules within 6 to 21 days after injection.

TABLE 3
Tumor Formation by Lymphocyte Clones

| | Experiment[a] | | | | | | | |
|---|---|---|---|---|---|---|---|---|
| Clone | I | II | III | IV | V | VI | VII | VIII |
| 1A9 | 2/2[b] | 2/2 | — | 2/2 | 2/2 | 2/2 | 12/12 | — |
| 2E8 | — | 2/2[c] | — | 0/2 | 0/2 | 0/2 | — | 0/2 |
| 2H6 | — | — | 0/2 | 0/2 | 0/2 | — | — | 0/2 |
| | (Nu) | (Nu) | (Nu) | (SCID) | (Nu) | (Nu) | (SCID) | (SCID) |

[a]Numbers of cells injected were $3 \times 10^7$ (Exper. I); $1–2 \times 10^6$ (Exper. II–IV); and $6 \times 10^3$–$1.5 \times 10^4$ (Exper. VII and VIII).
[b]All 1A9 tumors were always visible 6–21 days after inoculation.
[c]Tumors arose from 2E8 cells in this experiment only and were not visible before 7 weeks after inoculation. No tumors were detectable in subsequent experiments done with different freezings and observed for 8 weeks.

A striking finding was that the tumors contained large adherent cells with characteristics of stromal cells. In frozen sections of the 1A9 tumor examined with our KMA1.4 monoclonal antibody to stromal cells, a reticular staining pattern was obvious (not shown). Furthermore, it was possible to isolate stromal cells from this source, which have the capacity to support the clonal growth of lymphocyte clones in the methylcellulose assay.

The 1A9 cells readily migrated beneath stromal cells in culture, a phenomenon that has been termed pseudoemperipolesis (Nishi et al., 1982). This was also the case with cells recovered in primary cultures of 1A9 tumors. It is noteworthy that the growth of such tumor-derived 1A9 cells was, like the parent 1A9 cells, stromal-dependent. These observations indicate that 1A9 cells may attract and interact with stromal cells *in vivo*. It would be interesting to learn the origin of these stromal cells and whether they contribute in tumor cell growth.

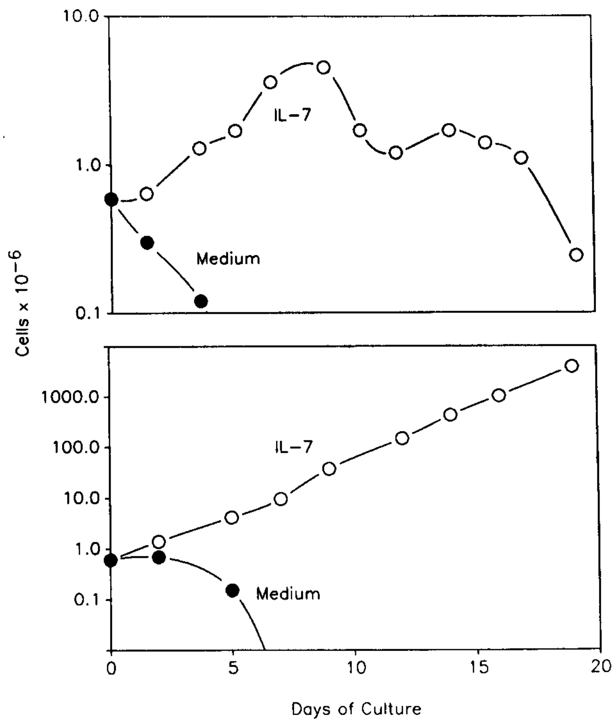

FIGURE 4. IL-7-dependent growth of lymphocyte clones. The top panel is typical of responses obtained when 2G5, 2H6, and 1A9 cells were removed from stromal-cell adherent layers and placed in liquid medium with IL-7. The lower panel, which utilizes a different scale, illustrates the continuous growth of 2E8 cells in this condition.

### Responsiveness to Cytokines

Interleukin-7 (IL-7) is a potent short-term stimulus for the growth of normal pre-B cells in culture (Lee et al., 1989). When assessed by thymidine incorporation in 72-h cultures, the growth of all of the lymphocyte clones was augmented by IL-7 (data not shown). The clones are, therefore, similar to freshly isolated bone marrow cells in this respect.

Continued addition of IL-7 to freshly isolated bone marrow cultures does not sustain cell division for more than a few weeks (Lee et al., 1989).

Similarly, most of our lymphocyte clones survived for only short periods when removed from stromal cells and cultured with this cytokine (Fig. 4). After an initial small increase in cell number, cell death appeared to balance cell division until survival could no longer be maintained. In contrast, the 2E8 lymphocyte clone expanded logarithmically when IL-7 was the only stimulus. This clone has been maintained in subculture with IL-7 for up to 8 months. The 2E8 cells responded to IL-7 in a dose-dependent fashion when evaluated with MTT or thymidine incorporation assays (not shown). The lower limit of cytokine concentration required for growth was similar to that reported previously for another IL-7-dependent lymphocyte clone (Namen et al., 1988; Park et al., 1990; and Namen, unpublished observations). A survey of other cytokines indicates that these cells can be exploited in IL-7 bioassays (Fig. 5).

The 1A9 clone was found to be capable of autonomous growth above a critical cell concentration (Fig. 6). This was not the case with any of our other lymphocyte clones, but is similar to one recently described in another laboratory (Lemoine et al., 1988). Those investigators obtained evidence for a low-molecular-weight cytokine that augmented growth at low cell densities. Autocrine growth stimulation may also occur with 1A9 cells and conditioned medium from any of the established clones, or a pre-B lymphoma cell line was stimulatory (Fig. 7). It is interesting that conditioned medium from BMS1 cells even had this effect, although 1A9 cells did not proliferate in direct contact with this stromal-cell clone (Fig. 3 and results not shown).

### DISCUSSION

The lymphocyte clones described here join many previously described hemopoietic cell lines that are informative with respect to differentiation events and the regulatory mechanisms that control them. These late pre-B and early B lymphocyte-stage cells have different requirements for survival and growth in culture. The most representative of normal bone marrow cells grow indefinitely on stromal cells and give a brief proliferative response to IL-7. An additional factor or factors are needed for their long-term propagation. One clone, which should be extremely valuable as a biological indicator, can grow continuously in this cytokine alone. Yet another lymphocyte clone, which was the only one consistently capable of tumor formation, is stromal-cell-dependent but responsive to autocrine signals. Thorough characterization of these lymphocyte clones with monoclonal antibodies and cDNA probes revealed that quantitative, rather than qualitative, changes in gene expression may occur as maturing cells make the transition from pre-B cells to B cells.

The coordinate influence of many microenvironmental stimuli provided by cytokines and contact with the extracellular matrix may be required for differentiation of multipotential stem cells to functional B lymphocytes. The lymphocyte clones described here are indicators of growth and survival factors only. Although two of them responded to interferon by expression of Ly-6A, there was little indication that the clones could undergo significant maturation. In the case of 1A9 cells, a variety of sur-

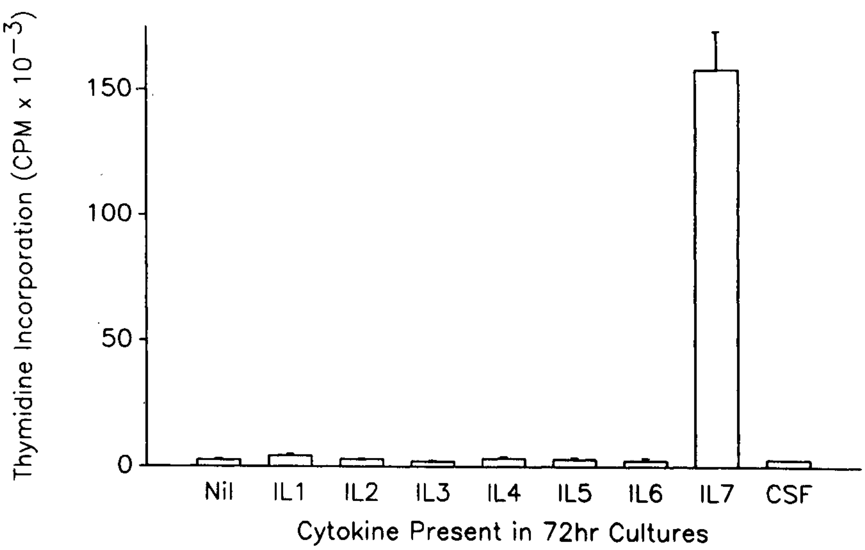

FIGURE 5. A survey of cytokines that might influence replication of 2E8 cells. The indicated cytokines were added to liquid medium at doses listed in Materials and Methods and proliferation was assessed 48 hr later by thymidine incorporation.

face and cytoplasmic markers was virtually unchanged even after recovery from nude mouse tumors.

Random mutations may occur in lymphocytes held in long-term culture to result in a simplification of their growth requirements. The 2E8 clone would seem to represent cells that retain only the need for IL-7, whereas normal lymphocytes and the other cloned lines respond only transiently to this factor (Lee et al., 1989). Although our survey revealed no other cytokines that stimulate 2E8 cells, they do grow on the S17 stromal-cell clone (Collins and Dorshkind, 1987), which is thought not to elaborate IL-7 (K. Dorshkind, C. Paige, and O. Witte, personal communications). Thus, 2E8 cells might be useful for identification of a novel cytokine(s) made by that stromal-cell clone. Similarly, 1A9 cells could serve as indicators for a seemingly ubiquitous stimulus, which may have previously been studied by other investigators (Lemoine et al., 1988; Muirhead and Davis, 1990).

It should be noted that only large pre-B cells, and not the small pre-B or newly formed B cells from bone marrow, can be stimulated by IL-7 (Lee et al., 1989). The 2E8, 2H6, and 2G5 clones are all phenotypically B cells, that is, are surface IgM-positive and thus past the stage of differentiation that is normally responsive to this cytokine. These clones also

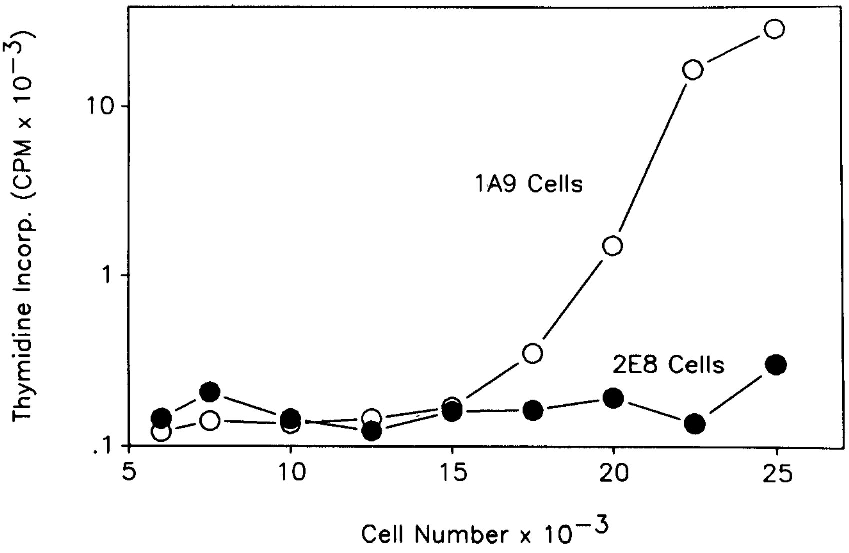

FIGURE 6. Autonomous growth of 1A9 cells when held at high initial cell densities. The 1A9 clone was unique in being able to survive and grow at high density in liquid medium. For thse experiments, cells were removed from stromal cells and passed through G-10 columns before use.

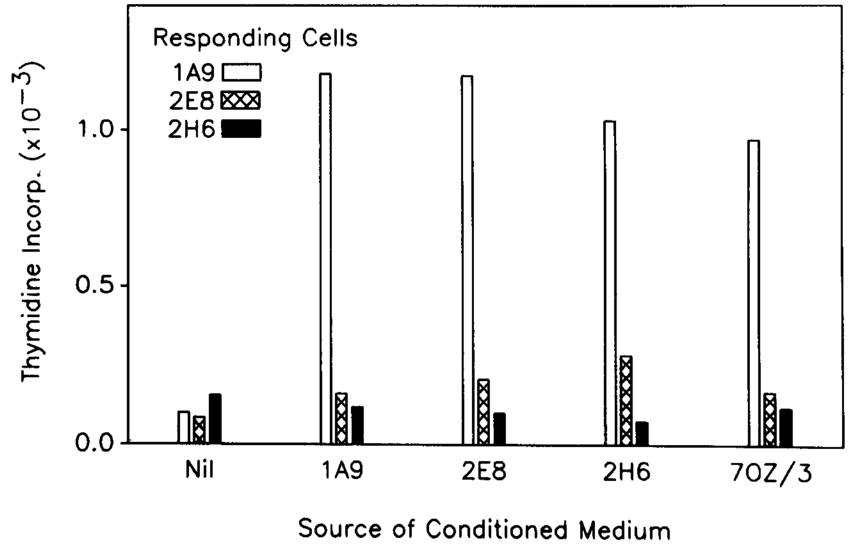

FIGURE 7. Responsiveness of 1A9 cells to conditioned medium from various cell lines. While at low initial cell densities, the 1A9 cells were stromal-cell-dependent; this requirement could be satisfied by conditioned medium from 1A9 cells grown at high density. This experiment was done with $6 \times 10^3$ cells/well and conditioned medium prepared as detailed in Materials and Methods.

differ from their normal counterparts in terms of cell-cycle status. The final stages in B lymphopoieses proceed with little or no cell division (Landreth et al., 1981). Therefore, IL-7 may promote the survival and continued proliferation of cells that are placed in cycle through other means. In this context, it is possible that IL-7 prevents programmed cell death, as has recently been suggested for other hemopoietic growth factors (Williams et al., 1990).

There are a number of common characteristics, as well as some heterogeneity, among the lymphocyte supporting stromal-cell lines that have been described (reviewed in Kincade, 1987; Kincade et al., 1989). In terms of function, all lymphocyte clones grew and could be cloned in methyl cellulose on the BMS2 stromal-cell clone. However, a more complex pattern of growth was obtained with other stromal cell lines (Fig. 2) and the molecular basis for this is not yet clear. The most noteworthy difference in the clones used for this study is their apparent ability to make IL-7 (Gimble et al., 1989b). Little or no mRNA for IL-7 was detected in preparations of our spleen-derived stromal cells and the lymphocyte clones did not grow well on them. However, we caution that levels of the transcript for this cytokine are always at the limit of detection and can fluctuate with time following subculture of stromal cells. Furthermore, at least one stromal-cell subclone can actively suppress lymphocyte growth (Kincade et al., 1990). Nishikawa and colleagues found that two of four stromal-cell-dependent lymphocyte clones grew on a stromal-cell clone that does not make, and cannot be induced to make, IL-7 (Nishikawa et al., 1988; Sudo et al., 1989). Until experiments with neutralizing antibodies are performed, we believe it unwise to attribute all proliferative activity to IL-7.

Our 1A9 cells share an interesting feature previously found in only some murine and human pre-B lymphoma lines (Paige et al., 1981; Hardy et al., 1986; Pillai and Baltimore, 1987; Kerr et al., 1989; Park et al., 1990). That is, detectable quantities of $\mu$ heavy chains are present on the cell surface in the absence of conventional light chians. As with the 70Z/3 lymphoma line, virtually all of the $\mu$ chains were associated with "surrogate" light chains, which on reducing gels were approximately 18 kD (Pillai and Baltimore, 1987). However, in contrast to 70Z/3 cells, which are inducible for expression of $\kappa$ (Paige et al., 1978), conventional light-chain genes are unrearranged in this new clone. At least one component of the surrogate light chains precipitated from 1A9 cells is a product of the $\lambda_5$ (Sakaguchi and Melchers, 1986; Tsubata and Reth, 1990) gene, for which mRNA was easily detectable on Northern blots. It is particularly noteworthy that some surrogate light chain was detectable on 2E8 cells, which rearranged, expressed, and displayed conventional light chain on the surface. Thus, differential expression of these two genes is not coordinately controlled in this line. In a previous study of Abelson-virus-transformed lymphocytes, acquisition of surface Ig also did not correspond to abrupt termination of pre-B-cell characteristics (Bender and Kuehl, 1987). Furthermore, another group has just found that loss of surrogate light chain did not accompany pre-B-cell maturation (Takemori et al., 1990).

It has recently been demonstrated that at least three proteins are associated with surface immunoglobulin on mature B cells (Haustein and Von der Ahe, 1986; Hombach et al., 1988; Campbell and Cambier, 1990; Chen et al., 1990; Hombach et al., 1990; Takemori et al., 1990; Wienands et al., 1990). These are most easily visualized by use of digitonin in the membrane-extraction procedure and a complex of 33–36 kD was detectable with our biotin-labeling procedure using the W279 B lymphoma line (Fig. 1B). Similar analysis of one pre-B- and one newly formed B-cell clone indicated that fewer Ig-associated species are displayed on the surface of these immature cells. It is possible that this difference is due to technical limitations in labeling low numbers of such molecules on the surface of pre-B cells and experiments are underway to address that possibility. Such Ig-associated proteins are thought to participate in signal transduction via antigen receptors on B cells similar to the function of the CD3 complex on T cells (Oettgen et al., 1986). At least one of them (MB 1; B34; IgM$\alpha$) is required for surface display of immunoglobulins on plasma cells (Hombach et al., 1990). Our findings suggest that only one may be sufficient for surface expression of $\mu$-plus surrogate light chain on late stage pre-B cells.

Our analyses of these lymphocyte clones provide the most detailed information yet available about cells that are undergoing the final events in B-cell formation. It might be expected that expression of many genes would abruptly change to facilitate migration from the bone marrow (adhesion molecules) and responsiveness to foreign antigen (Ig-associated proteins, tyrosine kinases, etc.). However, most of the changes that corresponded to the display of complete surface immunoglobulin molecules were quantitative. One exception to this pattern was the transmembrane proteoglycan Syndecan, which was detected on pre-B-, but not B-cell clones (Table 1). Further studies might reveal a unique function for Syndecan on maturing pre-B cells (Sanderson et al., 1989).

Lymphocytes grown in conventional long-term cultures only rarely form tumors and only after being maintained for longer than 6 months (Whitlock et al., 1984). Of the lymphocyte clones that we tested, only 1A9 was consistently tumorigenic. There is precedent for lymphocyte lines that depend on host-derived cytokines for tumor formation (Lasky and Thorbecke, 1989) and further studies might reveal the origin of stromal cells that are attracted to 1A9 tumors. The clone is also the best example we have found of lymphocytes that actively migrate beneath stromal cells in culture. The basis for this interesting phenomenon, known as "pseudoemperipolesis" (Witte et al., 1987), is unknown but could be explained in terms of chemotactic factors concentrated beneath stromal cells, or a polarization or redistribution of stromal-cell membranes.

We expect that these and other lymphocyte clones isolated from long-term bone marrow cultures will be valuable for detecting regulatory cytokines and for investigating stromal-cell functions. They may also be exploited in studies of the molecular events that occur as lymphoid cells progress from a normal to transformed phenotype. Finally, clones that span the pre-B- to B-cell transition stages provide an opportunity to explore mechanisms that control the surface display of surrogate light chains and other $\mu$-chain-associated proteins.

### MATERIALS AND METHODS

### Establishment and Maintenance of Lymphocyte Clones

Long-term bone marrow cultures from immunodeficient BALB/c.*xid* or normal BALB/c mice were prepared and maintained as described in our previous publications (Pietrangeli et al., 1988; Hayashi et al., 1989). Lymphocyte clones were derived by limiting dilution on stromal-cell layers prepared from CBA/HT6T6 marrow. They were routinely maintained throughout this study on stromal-cell clones or with recombinant IL-7. Generally, stromal cells (approximately $10^5$) were plated the night previously in 100-mm dishes (Corning) to yield subconfluent layers. Approximately $2\times10^6$ lymphocytes were added per dish in 8 ml of Whitlock-Witte medium. These cultures were fed with 75% fresh medium at weekly intervals and subcultured monthly to fresh adherent stromal-cell layers. The IL-7-dependent 2E8 clone was adapted to growth in this cytokine and maintained at an approximate concentration of $5\times10^5$ cells/ml with 1,000 units/ml of murine or 1 ng/ml of human recombinant IL-7 (PeptroTech, Inc., Rocky Hill, NJ). Cells were harvested at least every 3 days, and adjusted to the original cell concentration with fresh medium and IL-7. This cell density was critical to maintainence of satisfactory viability and growth of 2E8 cells, while oxygen tension (5% $O_2$ vs. ambient $O_2$) had no obvious influence. The initial derivation and cloning of 2E8 cells was done with Whitlock-Witte medium, but growth was more vigorous in McCoy's Modified 5A medium (Gibco) with 5% FCS (Hyclone, Logan Utah, Lot #1111885) $5\times10^{-5}$ M 2-mercaptoethanol, L-glutamine, and Penn-strep. Functional assessment of stromal cells was done by means of the methylcellulose colony assay previously described in detail (Pietrangeli et al., 1988). Initial numbers of stromal cells were $3\times10^4$ per 35-mm dish (Lux) and colonies were scored 7 days later with a Zeiss IDO3 inverted microscope with a 10× objective. Foci containing greater than 40 cells were counted as colonies.

### Cell-Surface-Marker Analysis

Immunofluorescence and flow cytometry were used to assess surface-marker expression on lymphocyte clones. Particularly valuable monoclonal antibodies were donated by Drs. J. Kearney ($\lambda$), M. Cooper (BP-1), M. Bernfield (Syndecan), and U. Hammerling (Ly-6). Anticlass II antibodies ($Ia^d$) were purchased from Becton-Dickinson. Responsiveness to inductive stimuli was assessed after 48 h of culture at a concentration of $6.25\times10^5$ cells per ml in 100-mm tissue-culture dishes (Corning). Either 500 units per ml of recombinant rat interferon gamma (Amgen) or 10 $\mu$g per ml of lipopolysaccharide were added to particular cultures. Expression of surface $\kappa$ and Ly-6 antigens was then assessed with a Coulter Epics V flow cytometer.

### Cell-Surface Labeling and Immunoprecipitation

Cells were surface-labeled by a novel biotinylation procedure (Miyake et al., 1990; Miyake, in preparation). Briefly, after three washes in HBSS, cells were suspended in labeling buffer containing 100 mM HEPES, 150 mM NaCl, pH 8.0, at a concentration of $1 \times 10^7$/ml. Sulfo-N-hydroxysuccinimidobiotin (sulfo-NHS-biotin; Pierce Chemical Co., Rockford, IL) was added to cell suspensions such that the final concentration of sulfo-NHS-biotin was 0.1 mg/ml. After a 30 min incubation at room temperature with occasional shaking, cells were washed once in chilled RPMI 1640 and twice in RPMI 1640 supplemented with 3% FCS. The cells were lysed for 15 min on ice at a concentration of $5 \times 10^7$/ml in Triton X-100 lysis buffer (1% Triton X-100; 10 mM Tris, pH 7.5, 1 mM phenyl methyl sulfonyl fluoride (PMSF), 10 $\mu$g/ml soybean trypsin inhibitor, 1 $\mu$g/ml leupeptin, 1 U/ml aprotinin, 50 mM iodoacetamide, 0.1% $NaN_3$, and 1 mM EDTA) or in digitonin lysis buffer (identical to the previous but with 1% digitonin (Sigma)). After centrifugation, the lysates were precleared three times with 40 $\mu$l of immobilized protein G agarose (Pierce) conjugated with normal rabbit serum. The precleared lysates were added to 40 $\mu$l of rabbit antimouse IgM (Miles) conjugated protein G agarose. The mixture was rotated for 60 min at 4°C and then washed three times with a 10 mM Tris-HCl buffer, pH 7.5, containing 150 mM NaCl, 0.1% $NaN_3$, and 0.2% Triton X-100, or 0.2% digitonin, respectively. The absorbed proteins were released by boiling for 5 min in a sample buffer containing 0.125 Tris-HCl, 2% SDS, 10% glycerol, and 5% 2ME, pH 6.8. Then SDS-PAGE electrophoresis was carried out on Laemmli 10–12% polyacrylamide gels.

### Blotting and Developing

After electrophoresis, gels were equilibrated for 30 min in transfer buffer (0.025 M tris, 0.192 M glycine, and 20% methanol). Proteins were then electrophorectically transferred to Trans-Blot membranes (Bio-Rad) for 1 hr at 100 V in a Trans-Blot cell (Bio-Rad). Membranes were then soaked for 1 hr in PBS containing 10% BSA, 0.05% Tween 20, 0.1% Thimerosal, followed by 1 hr of incubation in PBS containing 0.1% avidin-horseradish peroxidase, 1% BSA, and 0.05% Tween 20. After extensive washing in PBS with 0.05% Tween 20, membranes were developed in PBS containing 20% methanol, 0.6 mg/ml 4-chloro-1-napthol, and 0.003% hydrogen peroxide.

### Northern and Southern Blots

Northern blots were prepared and analyzed as in our previous publications (Gimble et al., 1989b; Gimble et al., 1990). The cDNA probes were generously donated by Drs. F. Melchers ($\lambda_5$); J. Parnes (Lyb-2); T. Bender (Myb); F. Alt (N-Myc); R. Wall (B29, B37, CHO-b, J$\kappa$, $JH_{3-4}$); M. Vakil ($V_\lambda 1$, $V_\lambda 2$); R. Perlmutter (Lck); S. Korsmeyer (BCL2); D. Baltimore (TdT); S. Goyert (CD14); S.-I. Hayashi (cKit); S. Clark (IL-6); and S. Gillis (IL-7). Southern blot analyses were done utilizing a method for small numbers of cells (Williams, 1987).

### Tumorigenicity

For the assessment of stromal-cell tumorigenicity, $1 \times 10^6$ cells in 200 $\mu$l of PBS without $Ca^{++}/Mg^{++}$ were injected subcutaneously into the backs of BALB/c.nu/nu or CB.17.SCID mice. As a control, similar numbers of NIH 3T3 cells were injected and this always resulted in tumor formation. In the case of lymphocyte clones, 1.5–2.0 $\times 10^6$ cells in 200 $\mu$l of PBS were injected subcutaneously. A PBS control was done for each experiment and the animals were checked for tumors biweekly for a period of 8 weeks.

### Tumor Cell Recovery

Animals were euthanized with $CO_2$ and submerged in 70% ethanol for 1 min. A 3-cc syringe with an 18G, 1½-in. needle was inserted into the tumor (the outer periphery) and tumor cells withdrawn. Two milliliters of media (RPMI 1640, 5% FCS) were then drawn into the syringe and the biopsy ejected into a sterile tube. A viable cell count was done using trypan blue dye exclusion and the cells were cultured in media consisting of RPMI 1640, 5% FCS, $5 \times 10^{-5}$ M 2 ME, L-glutamine, Penn/Strep at a concentration of $1 \times 10^6$ cells per ml. Within 7–10 days, adherent cells could be seen in the culture flasks.

### Frozen Sections

Small cubes of tumor tissue were immersed in O.C.T. compound (Miles Scientific) in plastic wells floating in 2 Methylbutane (Fisher) in a stainless steel beaker. The beaker was then placed in liquid nitrogen for 1 min or until the O.C.T. was almost completely frozen. Specimens were then stored in sealed bags at −20°C before sectioning on a microtome. Immunoperoxidase staining was performed as in our previous studies (Witte et al., 1987) with monoclonal KMA1.4 antibodies to stromal cells (K. Miyake, manuscript in preparation).

### Responsiveness to Cytokines

Survival and proliferation of lymphocytes were assessed by direct counting with Trypan blue due, by MTT assay (Mosmann, 1983), and by $^3$H thymidine incorporation (Pietrangeli et al., 1988). A survey was made to determine if the IL-7-dependent 2E8 clone is responsive to other recombinant cytokines. These included IL-1 (from Genzyme 100 U/ml), IL-2 (from Genzyme, 10 U/ml), IL-3 (donated by Immunex, 10–1,000 U/ml), IL-4 (Immunex, 20–100 U/ml), IL-5 (donated by Dr. K. Takatsu, 10–100 U/ml), IL-6 (donated by Genetics Institute, 1/10 dilution), and G/M-CSF (Immunex, 25–100 U/ml). As additional sources of CSFs, we tested conditioned medium prepared from L cells and WEHI-3 cells.

### Preparation of Conditioned Media

Confluent cultures of stromal cells were generated in 100-mm dishes and all medium was aspirated and replaced with 5 ml of Whitlock-Witte medium. After an additional 7 days of culture, conditioned medium was harvested and centrifuged at 5000 rpm for 10 min to remove cells and debris. Lymphocytes were harvested and washed four times with medium to remove residual cytokines. Those maintained on stromal-cell feeder layers were also passed through a G10 Sephadex column to remove any contaminating stromal cells. The cells were cultured at $5 \times 10^5$ cells per ml, 2 ml per well in Corning 24 well plates and conditioned medium collected after 48 h. The conditioned medium was centrifuged at 5000 rpm for 10 min to remove dead cells and debris. All conditioned mediums were stored at 4°C until assayed at final concentrations of 10–25% total volume.

### ACKNOWLEDGMENTS

This work was supported by grants AI-20069 and AI-19884 from the National Institute of Health. We thank Drs. Linda Thompson and Carol Webb for helpful comments on the manuscript.

*(Received ?)*

*(Accepted October 12, 1990)*

### REFERENCES

Bender T.P., and Kuehl W.M. (1987). Differential expression of the c-*myb* proto-oncogene marks the pre-B cell/B cell junction in murine B lymphoid tumors. J. Immunol. **139**: 3822–3827.

Campbell K.S., and Cambier J.C. (1990). B lymphocyte antigen receptors (mIg) are noncovalently associated with a disulfide linked, inducibly phosphorylated glycoprotein complex. EMBO J. **9**: 441–448.

Chen J., Stall A.M., Herzenberg L.A., and Herzenberg L.A. (1990). Differences in glycoprotein complexes associated with IgM and IgD on normal murine B cells potentially enable transduction of different signals. EMBO J. **9**: 2117–2124.

Collins L.S., and Dorshkind K. (1987). A stromal cell line from myeloid long-term bone marrow cultures can support myelopoiesis and B lymphopoiesis. J. Immunol. **138**: 1082–1087.

Cooper M.D., Mulvaney D., Coutinho A., and Cazenave, P.-A. (1986). Identification of a novel cell surface molecule on early B-lineage cells. Nature **321**: 616–618.

Desiderio S.V., Yancopoulos G.D., Paskind M., Thomas E., Boss M.A., Landau N., Alt F.W., and Baltimore D. (1984). Insertion of N regions into heavy-chain genes is correlated with expression of terminal deoxytransferase in B cells. Nature **311**: 752–755.

Dymecki S.M., Niederhuber J.E., and Desiderio S.V. (1990). Specific expression of a tyrosine kinase gene, blk, in B lymphoid cells. Science **247**: 332–336.

Garvin A.M., Pawar S., Marth J.D., and Perlmutter R.M. (1988). Structure of the murine *lck* gene and its rearrangement in a murine lymphoma cell line. Mol. Cell. Biol. **8**: 3058–3064.

Gimble J.M., Dorheim M.A., Cheng Q., Pekala P., Enerback S., Ellingsworth L., Kincade P.W., and Wang C.-S. (1989)a. Response of bone marrow stromal cells to adipogenetic antagonists. Mol. Cell. Biol. **57**: 4587–4595.

Gimble J.M., Pietrangeli C.E., Henley A., Dorheim M.A., Silver J., Namen A.E., Takeichi M., Goridis C. and Kincade P.W. (1989b). Characterization of murine bone marrow and spleen derived stromal cells: Analysis of leukocyte marker and growth factor mRNA transcript levels. Blood **74**: 303–311.

Gimble J.M., Dorheim M.-A., Cheng Q., Medina K., Wang C.-S., Jones R., Koren E., Pietrangeli C.E., and Kincade P.W. (1990). Adipogenesis in a murine bone marrow stromal cell line capable of supporting B lymphocyte growth and proliferation: Biochemical and molecular characterization. Eur. J. Immunol. **20**: 379–387.

Hardy R.R., Dangl J.L., Hayakawa K., Jager G., and Herzenberg L.A. (1986). Frequent λ light chain gene rearrangement and expression in a Ly-1 B lymphoma with a productive λ chain allele. Proc. Natl. Acad. Sci. USA **83**: 1438–1442.

Haustein D., and Von der Ahe D. (1986). A 30-kDa protein is disulfide linked to IgM on normal and neoplastic murine B lymphocytes. Eur. J. Immunol. **16**: 113–115.

Hayashi S.-I., Witte P.L., and Kincade P.W. (1989). The Xid mutation affects hemopoiesis in long term cultures of murine bone marrow. J. Immunol. **142**: 444–451.

Hermanson G., Eisenberg D., Kincade P.W., and Wall R. (1988a). A new member of the immunoglobulin superfamily exclusively expressed on B lineage cells. Proc. Natl. Acad. Sci. USA **85**: 6890–6894.

Hermanson G., Law R., Davis M., Cohen D., Kincade P.W., and Wall R. (1988b). Isolation of lineage specific genes of B lymphocytes. *In:* B Cell Development. U.C.L.A. Symposia on Molecular and Cellular Biology, New Series, Witte, O., Howard M., and Klinman N., Eds. (New York: Alan R. Liss, Inc.) pp. 133–146.

Hombach J., Leclercq L., Radbruch A., Rajewsky K., and Reth M. (1988). A novel 34-kd protein co-isolated with the IgM molecule in surface IgM-expressing cells. EMBO J. **7**: 3451–3456.

Hombach J., Tsubata T., Leclercq L., Stappert H., and Reth M. (1990). Molecular components of the B-cell antigen receptor complex of the IgM class. Nature **343**: 760–762.

Kerr W.G., Cooper M.D., Feng L., Burrows P.D., and Hendershot L.M. (1989). Mu heavy chains can associate with a psuedo-light chain complex (Psi Lambda) in human pre-B cell lines. Int. I. **1**: 354–361.

Kincade P.W. (1987). Experimental models for understanding B

lymphocyte formation. Adv. Immunol. **41:** 181–267.

Kincade P.W., Lee G., Watanabe T., Sun L., and Scheid M.P. (1981). Antigens displayed on murine B lymphocyte precursors. J. Immunol. **127:** 2262–2268.

Kincade P.W., Lee G., Pietrangeli C.E., Hayashi S.-I., and Gimble J.M. (1988). Cells and molecules that regulate B lymphopoiesis in bone marrow. Annu. Rev. Immunol. **7:** 111–143.

Kincade P.W., Medina K., Pietrangeli C.E., Hayashi S.-I., and Namen A.E. (1990). Stromal cell lines which support lymphocyte growth. II. Characteristics of a suppressive subclone.

Gupta S., Paul W., Cooper M., and Rothenberg E. (Eds.) Mechanisms of lymphocyte activation and immune regulation III: Developmental Biology of Lymphocytes. New York: Plenum Press, 1990.

Kudo A., and Melchers F. (1987). A second gene, $V_{preB}$ in the $\lambda_5$ locus of the mouse, which appears to be selectively expressed in pre-B lymphocytes. EMBO J. **6:** 2267–2272.

Landreth K.S., Rosse C., and Clagett J. (1981). Myelogenous production and maturation of B lymphocytes in the mouse. J. Immunol. **127:** 2027–2034.

Lasky J.L., and Thorbecke G.J. (1989). Characterization and growth factor requirements of SJL lymphomas. II. Interleukin 5 dependence of the *in vitro* cell line, cRCS-X, and influence of other cytokines. Eur. J. Immunol. **19:** 365–371.

Lee G., Namen A.E., Gillis S., Ellingsworth L.R., and Kincade P.W. (1989). Normal B cell precursors responsive to recombinant murine IL-7 and inhibition of IL-7 activity by transforming growth factor-$\beta$. J. Immunol. **142:** 3875–3883.

Lemoine F.M., Krystal G., Humphries R.K., and Eaves C.J. (1988). Autocrine production of pre-B-cell stimulating activity by a variety of transformed murine pre-B cell lines. Cancer Res. **48:** 6438–6443.

Miyake K., Medina K.L., Hayashi S.-I., Ono S., Hamaoka T., and Kincade P.W. (1990). Monoclonal antibodies to Pgp-1/CD44 block lympho-hemopoiesis in long-term bone marrow cultures. J. Exp. Med. **171:** 477–488.

Mosmann T. (1983). Rapid colorimetric assay for cellular growth and survival: Application to proliferation and cytotoxicity assays. J. Immunol. Methods **65:** 55–63.

Muirhead M.J., and Davis R.T. (1990). A cloned bone marrow-drived stromal cell line elaborates a dialyzable lymphopoietic co-factor. J. Cell. Biochem. **S14D:** 207.

Nakayama E., Von Hoegen I., and Parnes J.R. (1989) Sequence of the Lyb-2 B-cell differentiation antigen defines a gene superfamily of receptors with inverted membrane orientation. Proc. Natl. Acad. Sci. USA **86:** 1352–1356.

Namen A.E., Schmierer A.E., March C.J., Overell R.W., Park L.S., Urdal D.L., and Mochizuki D.Y. (1988). B cell precursor growth-promoting activity. Purification and characterization of a growth factor active on lymphocyte precursors. J. Exp. Med. **167:** 988–1002.

Nishi Y., Yoshikawa K., Hiai H., Notake K., Shisa H., and Nishizuka Y. (1982). Formation of symbiotic complex by microenvironment-dependent mouse leukemias and thymic epithelial reticular cells. JNCI **69:** 627–633.

Nishikawa S.-I., Ogawa M., Nishikawa S., Kunisada T., and Kodama H. (1988). B lymphopoiesis on stromal cell clone: Stromal cell clones acting on different stages of B cell differentiation. Eur. J. Immunol. **18:** 1767–1771.

Oettgen H.C., Pettey C.L., Maloy W.L. and Terhorst C. (1986). A T3-like protein complex associated with the antigen receptor on murine T cells. Nature **320:** 272–275.

Paige C.J., Kincade P.W., and Ralph P. (1978). Murine B cell leukemia line with inducible surface immunoglobulin expression. J. Immunol. **121:** 641–647.

Paige C.J., Kincade P.W., and Ralph P. (1981). Independent control of immunoglobulin heavy and light chain expression in a murine pre-B cell line. Nature **292:** 631–633.

Park L.S., Friend D.J., Schmierer A.E., Dower S.K., and Namen A.E. (1990). Murine interleukin-7 (IL-7) receptor. Characterization on an IL-7-dependent cell line. J. Exp. Med. **171:** 1073–1089.

Park Y.H., and Osmond D.G. (1987). Phenotype and proliferation of early B lymphocyte precursor cells in mouse bone marrow. J. Exp. Med. **165:** 444–458.

Pietrangeli C.E., Hayashi S.-I., and Kincade P.W. (1988). Stromal cell lines which support lymphocyte growth: Characterization, sensitivity to radiation and responsiveness to growth factors. Eur. J. Immunol. **18:** 863–872.

Pillai S., and Baltimore D. (1987). Formation of disulphide-linked $\mu_2\omega_2$ tetramers in pre-B cells by the 18K $\omega$-immunoglobulin light chain. Nature **329:** 172–174.

Reth M. (1989). Antigen receptor tail clue. Nature **338:** 383–384.

Sakaguchi N., and Melchers F. (1986). $\lambda_5$, a new light-chain-related locus selectively expressed in pre-B lymphocytes. Nature **324:** 579–582.

Sanderson R.D., Lalor P., and Bernfield M. (1989). B lymphocytes express and lose syndecan at specific stages of differentiation. Cell Regulation **1:** 27–35.

Sudo T., Ito M., Ogawa Y., Iizuka M., Kodama H., Kunisada T., Hayashi S.-I., Ogawa M., Sakai K., Nishikawa S., and Nishikawa S.-I. (1989). Interleukin-7 production and function in stromal cell-dependent B cell development. J. Exp. Med. **170:** 333–338.

Takemori T., Mizuguchi J., Miyazoe I., Nakanishi M., Shigemoto K., Kimoto H., Shirasawa T., Maruyama N., and Taniguchi M. (1990). Two types of $\mu$ chain complexes are expressed during differentiation from pre-B to mature B cells. EMBO J. **9:** 2493–2500.

Thomas M.L., Reynolds P.J., Chain A., Ben-Neriah Y., and Trowbridge I.S. (1987). Proc. Natl. Acad. Sci. USA **84:** 5360–5363.

Tominga A., Mita S., Kikuchi Y., Hitoshi Y., and Takatsu K. (1989). Establishment of IL-5 dependent early B cell lines by long-term bone marrow cultures. Growth Factors **1:** 135–146.

Tsubata T., and Reth M. (1990). The products of the pre-B cell-specific genes ($\lambda_5$ and $V_{preB}$) and the immunoglobulin $\mu$ chain form a complex that is transported onto the cell surface. J. Exp. Med. **172:** 973–976.

Whitlock C.A., Robertson D., and Witte O.N. (1984). Murine B cell lymphopoiesis in long-term culture. J. Immunol. Methods **67:** 353–369.

Wienands J., Homback J., Radbruch A., Riesterer C., and Reth M. (1990). Molecular components of the B cell antigen receptor complex of class IgD differ partly from those of IgM. EMBO J. **9:** 449–455.

Williams G.T. (1987). High-efficiency preparation of DNA from limiting quantities of eukaryotic cells for hybridization analysis. Gene **53:** 121–126.

Williams G.T., Smith C.A., Spooncer E., Dexter T.M., and Taylor D.R. (1990). Haemopoietic colony stimulating factors promote cell survival by suppressing apoptosis. Nature **343:** 76–79.

Witte P.L., Kincade P.W., and Vetvicka V. (1986). Interculture variation and evolution of B lineage lymphocytes in long-term bone marrow culture. Eur. J. Immunol. **16:** 779–788.

Witte P.L., Robinson M., Henley A., Low M.G., Stiers D.L., Perkins S., Fleischman R.A., and Kincade P.W. (1987). Relationships between B-lineage lymphocytes and stromal cells in long term bone marrow cultures. Eur. J. Immunol. **17:** 1473–1484.

Wu Q., Lahti J.M., Air G.M., Burrows P.D., and Cooper, M.D. (1990). Molecular cloning of the murine BP-1/6C3 antigen: A member of the zinc-dependent metallopeptidase family. Proc. Natl. Acad. Sci. USA **87:** 993–997.

Zimmerman K.A., Yancopoulos G.D., Collum R.G., Smith R.K., Kohl N.E., Denis K.A., Nau M.M., Witte O.N., Toran-Alerand D., Gee C.E., Minna J.D., and Alt F. (1986). Differential expression of *myc* family genes during murine development. Nature **319:** 780–783.

